# Dairy Farmers' Knowledge, Attitudes, and Practices (KAP) Towards Aflatoxin Contamination in Milk and Feeds in Bahir Dar, Ethiopia

**DOI:** 10.1155/2024/5568286

**Published:** 2024-10-23

**Authors:** Sosina Dires Sewunet, Elias Kebede, Achenef Melaku, Andnet Yirga Assefa, Atnaf Alebie, Aschalew Assefa, Habtamu Ayalew, Girma Birhan, Ambaye Worku Kenubih

**Affiliations:** ^1^College of Veterinary Medicine and Animal Sciences, University of Gondar, P.O. Box 196, Gondar, Ethiopia; ^2^Department of Veterinary Science, College of Agriculture and Environmental Science, Bahir Dar University, Bahir Dar, Ethiopia

## Abstract

Aflatoxins, primary foodborne mycotoxins, come from *Aspergillus flavus* and *Aspergillus parasiticus* fungi. They pose significant health risks to humans and animals, creating a major challenge in the dairy sector. The objective of this study is to evaluate the knowledge, attitudes, and practices (KAP) of dairy farmers regarding aflatoxin contamination in milk and feeds. Conducted as a cross-sectional study in Bahir Dar city between November 2019 and February 2020, this investigation randomly selected 106 dairy farms for data collection. Face-to-face interviews, facilitated by a semistructured questionnaire, were employed. Findings indicate that 59.4% of respondents displayed good knowledge, while a substantial 94.3% exhibited a favorable attitude. Intriguingly, only 1.9% implemented good practices. Notably, the educational background of dairy farmers emerged as a significant factor influencing their KAP (*p* < 0.05). Conversely, various sociodemographic factors did not yield a significant impact on the KAP of dairy farmers. Despite a robust knowledge base and favorable attitudes towards aflatoxin among dairy farmers, the study highlights a significant gap in the implementation of recommended practices. This finding emphasizes the necessity for increased efforts to cultivate and reinforce good practices. Collaborative initiatives involving diverse stakeholders are crucial to reducing aflatoxin contamination in the dairy industry.

## 1. Introduction

Aflatoxins are the most hazardous foodborne toxins globally, causing acute and chronic aflatoxicosis in humans and animals depending on the dosage. They are secondary metabolites produced by several Aspergillus species, notably *Aspergillus flavus* and *Aspergillus parasiticus* [1–4]. Aflatoxins are divided into four primary types according to their fluorescence under blue or green light: aflatoxin B1 (AFB1), aflatoxin B2 (AFB2), aflatoxin G1 (AFG1), and aflatoxin G2 (AFG2) [5, 6]. Contamination of food and feeds with these toxins has gained worldwide attention due to their severe health impacts, including liver cancer, growth stunting, immune suppression at low concentrations, and rapid death at high exposures [1, 7, 8].

In dairy farming, ensuring the safety of animal feed is crucial for overall food safety. Aflatoxins and their derivatives in milk and dairy products (e.g., cheese and cottage cheese) pose significant health risks as they are resistant to pasteurization and processing [9]. It is estimated that one-quarter of the global food and feed supply is contaminated by aflatoxins and other mycotoxins along the food chain. Animal feeds, typically composed of agricultural products such as cereal grains and oilseed cakes, can also include cereal grain stovers, grass silage, brewers' spent grain, stale bread, and kitchen and bakery wastes [6, 10]. These waste products are often tainted with fungi, contributing to mycotoxin contamination in cattle feed [3].

When cows ingest feed contaminated with AFB1, it is biotransformed into its hydroxylated metabolite, aflatoxin M1 (AFM1), which is then secreted in their milk. The level of AFM1 in milk is directly connected to the amount of AFB1 in the animal's feed. Studies indicate that approximately 0.3%–6.2% of ingested AFB1 is converted into AFM1 in milk [5, 11]. Aflatoxins are heat-resistant and remain stable during most processing treatments, which results in their presence in all milk products. The International Agency for Research on Cancer (IARC) has classified AFM1 as a carcinogen [6, 12, 13].

Beyond health effects, aflatoxins impose an economic burden on farmers, traders, and nations due to revenue losses from strict regulations that limit the sale of contaminated crops [1]. While AFB1 contamination was initially thought to be a problem primarily in developing countries, climate change has increased the risk in industrialized nations, including Europe [1, 13]. Over 80 countries regulate aflatoxins, with legal limits for AFM1 varying by geography, agricultural practices, and climate. These limits are subject to change and differ by country or region, impacting public health and socioeconomic development [5, 6, 8, 14–18].

As reviewed by [6], recent studies from Africa have reported high mean levels of AFM1 in milk samples from Ethiopia, with a maximum of 0.97 mg/L [19–21]. In contrast, studies in Europe did not indicate mean AFM1 levels higher than 0.05 mg/L [22]. A study from Morocco also showed AFM1 levels below EU limits in all milk and milk powder samples [23]. Ethiopia is favorable for the growth of aflatoxigenic fungi and hence the contamination of grains by aflatoxins. According to a study by [24] in Addis Ababa, Ethiopia, large milk sheds had highly contaminated milk with AFM1 in the range of 0.028 and 4.98 mg/L. However, the aflatoxin contamination limit of dairy and beef cattle feeds in Ethiopia is not standardized [24].

Furthermore, the impact of aflatoxin ingestion through contaminated feed extends to animal products. Given that milk is a staple in the diets of infants and young children in the area under study, concerns regarding aflatoxin contamination become particularly pronounced [10, 25–27]. Various studies across Ethiopia have consistently highlighted significant aflatoxin contamination as a persistently hot issue that needs further investigation in agricultural products. Despite the livestock sector's importance to the Ethiopian economy and livelihoods, productivity remains low due to inefficient feed, poor genetic material, and inadequate veterinary services. Feed shortages, substandard feed quality, environmental conditions, contamination, and traditional storage practices exacerbate livestock production challenges and aflatoxin damage. It is hypothesized that contributing factors include a lack of knowledge, inadequate regulations, and poor management practices. This study aims to assess and gather information on the current knowledge, attitudes, and practices (KAP) of dairy farmers towards aflatoxin contamination in feeds and milk.

## 2. Materials and Methods

### 2.1. Study Area

**Figure 1:**
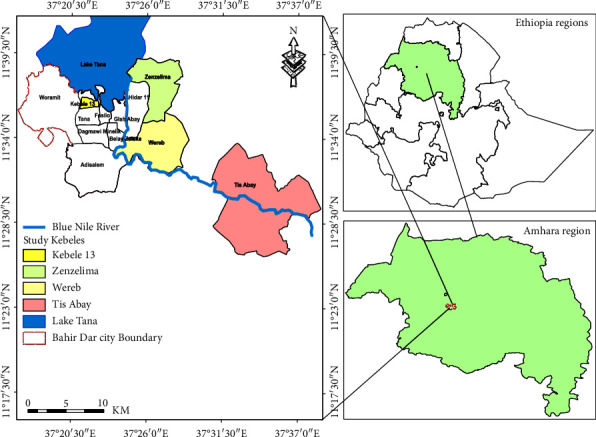
Map of the study area.

The research was conducted in four kebeles (Zenzelma, Kebele 13, Wereb, and Tise Abay) within the city of Bahir Dar as shown in Figure 1, situated approximately 565 km northwest of Addis Ababa, Ethiopia's capital. Positioned at 11°38′N latitude and 37°10′E longitude, the city boasts an average elevation of 1801 m above sea level. The climate is characterized by an average daily minimum and maximum temperature of 7°C and 29°C, respectively. The annual rainfall averages 1445 mm, with approximately 84% occurring between June and September.

Bahir Dar was intentionally chosen due to its pivotal role in the supply of animal feed to various production sites within the region, contributing to the overall production of milk and milk products. The selection of four kebeles was purposively due to the fact that these areas harbor a higher concentration of dairy farms compared to other kebeles in the city. This strategic selection enhances the study's relevance and applicability to the broader context of dairy farming in the region.

### 2.2. Study Design, Sample Size, and Sampling Technique

A cross-sectional study was conducted from November 2019 to February 2020 to assess the KAP of dairy farmers. Comprehensive lists of dairy farm owners in the four kebeles found under Bahir Dar city administration were prepared as sampling frame which were 787 dairy farm owners. Several project training directories, Bahir Dar city agricultural and animal health government office, and agency records were consulted in the preparation of the sampling frame to obtain the most comprehensive lists possible and to minimize selection bias. The approximate sample size for dairy farmers was determined from expected prevalence of 50% with defined precision of 10% and level of confidence of 95%. Hence, the sample size was calculated as it has been stated by the authors of [28].

(1) $$\begin{array}{c} n=\frac{Z\alpha^{2}xpxq}{L^{2}}, \end{array}$$

*n* = (1.96)2 (0.5 ∗ 0.5)/(0.1)2, *n* = (3.842) ∗ (0.25)/0.01, *n* = 0.9605/0.01 = 96.05—Then multiplied by 10% nonresponse rate. 96 ∗ 10% = 9.6—Round up to 10. *n* = 96 + 10 = 106—The total number of participants or dairy farm owners. Where *n* is the required sample size. *Zα* is confidence level: 95%, which corresponds to 1.96. *p* is proportion of the population: 0.5 (for maximum variability). *q* is complement of proportion: one- *p* = 0.5. *L* is margin of error: 0.1 (10%).

The respondents (106) were then randomly selected using a numbered list and a random number generator. Semistructured questionnaires were used to obtain more information. The questionnaires focused on the handling and storage of dairy feed, animal feeding, milk contamination, fungi growth in the feed, and their effects on the health of animals and humans. The questionnaires were developed and reviewed based on information collected from the literature and what the dairy farmers were practicing and approved by the research and ethical committee of the University of Gondar. The questionnaires were first prepared in English and later translated into Amharic (the local language).

### 2.3. Data Analysis

The statistical analysis for this study utilized SPSS software version 20. A one-way analysis of variance (ANOVA) and Chi-square (*χ*^2^) test of contingencies were used to analyze all the data. A *p* value of less than 0.05 (*p* < 0.05) was considered to show statistical significance.

For the evaluation of knowledge regarding aflatoxin contamination in feed and milk, a 15-point scale was employed. Each correct response was assigned a score of one, while incorrect responses received a score of zero. The total score ranged from zero to 15. The modified Bloom cutoff points, derived from Ms. Nahida's KAP Study 2007, were applied for interpretation. Score of 80%–100% was considered indicative of good knowledge, 50%–79% as satisfactory, and less than 50% as poor knowledge. Therefore, the scores with their respective knowledge levels were good knowledge (12–15), satisfactory knowledge (8–11), and poor knowledge (0–7).

Attitude was assessed through five Likert scale questions, capturing both positive and negative responses. Each correct response received a score of one, and incorrect responses were scored as zero. Total scores were calculated for each participant, and the mean score determined favorable or unfavorable attitudes towards aflatoxicosis. Scores above or equal to the mean were considered favorable (3–5), while scores below the mean indicated unfavorable (0–2) attitudes.

The practice score, ranging from zero to 12, was determined similarly. Correct responses were assigned a score of one, while incorrect responses got zero. A score of 80%–100% denoted good practice, 50%–79% indicated satisfactory practice, and less than 50% suggested poor practice. Therefore, the scores with their respective practice levels were good practice (9–12), satisfactory practice (6–8), and poor practice (0–5). This scoring system facilitated a comprehensive assessment of participants' KAP related to aflatoxin contamination.

### 2.4. Ethics Statement

This study was conducted in accordance with ethical standards. Informed consent was obtained from all participants in the farm prior to their involvement in the study. Participants were assured of the confidentiality of their responses, and their participation was voluntary. The research protocol was reviewed and approved by the Research and Ethical Committee of University of Gondar, Faculty of Veterinary Medicine and Animal Sciences, ensuring compliance with ethical guidelines and regulations.

## 3. Results

### 3.1. Sociodemographic Characteristics of the Respondents

The majority of the respondents was between the ages of 30 and 50 and was mainly milk producers, 97.2%. About 80.2% of the respondents were male. The other dominant sociodemographic characteristics of the respondents were farmers (64.2%), married (85.6%), small-scale dairy farm (96.2%), and a person who did not receive training (72.6%) as shown in Table 1.

### 3.2. KAP of the Dairy Farmer on Aflatoxin Contamination of Feed and Milk

**Figure 2:**
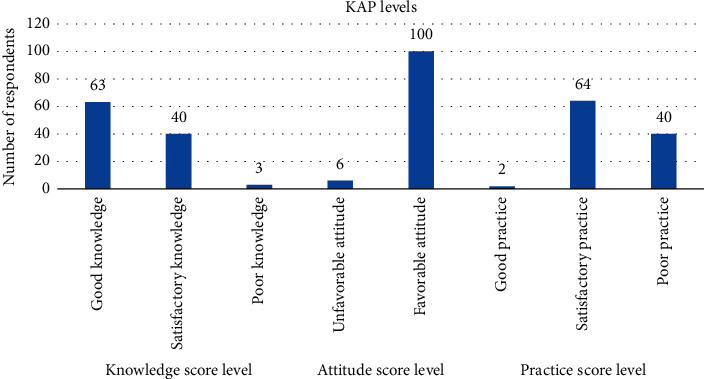
KAP levels of the respondents on aflatoxin contamination.

The findings from the KAP assessment revealed that 59.4% of the respondents possessed awareness about aflatoxin. Notably, a substantial majority (94.3%) exhibited a favorable attitude towards aflatoxin contamination. However, when it comes to practical application, despite the majority having good knowledge, only a minimal 1.9% of the respondents demonstrated a commendable level of practices. The graphical representation in Figure 2 underscores that the predominant trend among respondents was towards satisfactory and poor practices, highlighting a notable disparity between knowledge acquisition and its effective implementation in real-world practices.

Table 2 shows that the mean knowledge score of the respondents with a tertiary education level (12.20 ± 1.924) was significantly higher than that of the respondents with an educational level of secondary (10.85 ± 2.444), primary (12.16 ± 1.788), and illiterate (11.75 ± 1.481). Respondents within the age range of 30–50 years (12.09 ± 1.604) had a higher knowledge score compared to those respondents within 20–30 (10.43 ± 2.563) and over 50 years (11.82 ± 1.362). The knowledge score of the respondents who were owners of small-scale dairy farms (11.89 ± 1.659) was significantly higher than that of the respondents who had large-scale dairy farms (9.50 ± 3.109). However, there were no significant differences in aflatoxin knowledge scores between male and female respondents (*p* = 0.346), occupation (*p* = 0.730), marital status (*p* = 0.478), role in the food chain (*p* = 0.998), year of establishment of the farm (*p* = 0.277), and training (*p* = 0.288).

The respondents to the attitude score on aflatoxin contamination also varied significantly among illiterate (3.39 ± 0.695), primary (3.54 ± 0.869), secondary (3.92 ± 0.954), and tertiary education (3.20 ± 1.483) (Table 2). Respondents within the age range of 20–30 years (3.71 ± 1.267) had a higher attitude score than those respondents between 30 and 50 years (3.38 ± 0.787) and over 50 years (3.68 ± 0.670). Government employees (4.00 ± 0.001) and private employees (3.90 ± 1.071) had significantly higher attitude scores than housewives (3.69 ± 0.751), farmers (3.37 ± 0.771), and students (3.00 ± 0.001). The result showed that there were no significant differences in attitude scores for aflatoxin between other sociodemographic factors such as gender (*p* = 0.886), dairy farm (*p* = 0.196), marital status (*p* = 0.706), role in the food chain (*p* = 0.514), year of establishment of the farm (*p* = 0.338), and training (*p* = 0.171).

Table 2 shows that the practice scores on aflatoxin differed significantly between the type of dairy farm (*p* ≤ 0.001) and the level of education (*p* = 0.027). Significantly higher practice scores were reported in respondents at the secondary education level (6.54 ± 1.808) than in those at the primary (5.84 ± 1.214), illiterate (5.53 ± 1.419), and tertiary education levels (4.60 ± 2.302). Large-scale dairy farm owners (5.75 ± 3.862) had higher aflatoxins practice scores than small-scale dairy farm owners (5.72 ± 1.360). There were also no significant differences in aflatoxin practice scores between age groups (*p* = 0.317), male and female respondents (*p* = 0.694), occupation (*p* = 0.069), marital status (*p* = 0.514), role in the food chain (*p* = 0.999), year of establishment (*p* = 0.371), or training (*p* = 0.052).

**Figure 3:**
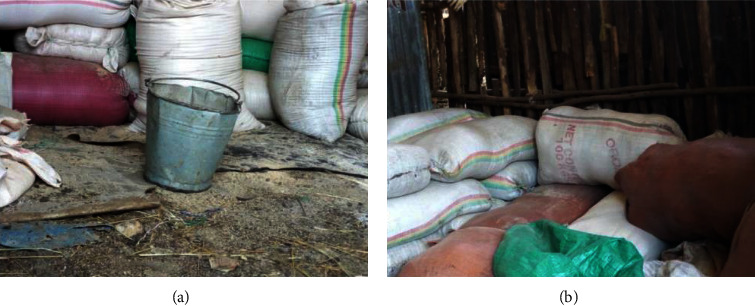
Feed storage conditions observed in the study area: unclean and not dried floors of the storage room (a) and walls of the storage room without rodent and insect control (b).

**Figure 4:**
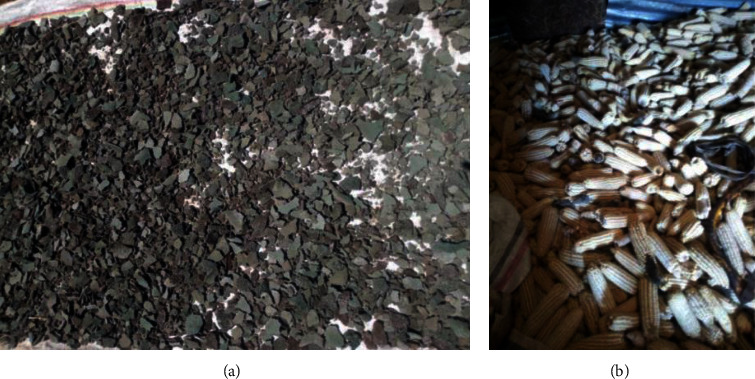
Possible source of contaminated feeds in the study area: moldy injera prepared for making “Tella,” which was later used as feed for cows (a) and moldy and deteriorated maize selected and stored as animal feed (b).

The research findings indicate that almost the majority of dairy farmers do not have good practices, which is also shown in Figures 3(a) and 3(b), and they store feed under poor conditions which is uncleaned and no pest control measures to prevent pests from introducing moisture or fungi into the feed. It can have a detrimental impact on both the quality of the feed and the health of the animals. In the images provided, we can see in Figure 4 moldy injera (a traditional Ethiopian bread) and moldy and deteriorated maize (b) that have been selected and stored as animal feed.

## 4. Discussion

The current study aimed to assess the KAP regarding aflatoxin contamination among dairy farmers. The findings indicated majority of participants were aged between 30 and 50 years (60.4%), with a smaller percentage aged 20–30 years (13.2%). This differs from a study in [29], where a significant portion of respondents were younger, aged 18–25 years (47.3%), and only 14.8% were aged 36–45 years. Similarly, a study in Cameroon reported that 48% of participants were aged 30–45 years [30]. In contrast, a study done in the southern part of Ethiopia [19] found that 89.1% of respondents were aged 18–33 years, indicating a younger respondent compared to this study. Another study in central Ethiopia [31] reported that 79.8% of respondents were older than 51 years, suggesting a broader age range in different regions.

In terms of gender, the present study had a predominance of male participants (80.2%). This is higher than in the Ghanaian studies [29, 32], where males constituted 52.3% and 41.1%, respectively. Conversely, a study in Cameroon found an almost equal gender distribution, with females making up 50.3% [30]. The [19] study reported 92% female participants, indicating a significant gender difference compared to the current study. Another study in [31] showed 57.4% female participants, again highlighting regional variations.

Educational levels among participants in this research result showed a high percentage of illiterate respondents (48.1%) and those with only primary education (34.9%), while only 4.7% had tertiary education. This contrasts sharply with the studies [30, 32], where 47.6% and 80.4% of respondents had tertiary education, respectively, while other study conducted in Pakistan reported only 16.9% farmers had graduation or higher degrees [14]. This suggests that the educational background of participants in the current study is lower compared to some other regions.

Regarding the type of dairy farming, the current study indicated that 96.2% of participants were involved in large-scale farming. This is different from findings in Cameroon, where the majority of participants were small-scale farmers [30]. This discrepancy could be attributed to regional differences in farming practices and economic conditions. In this study, 85.8% of respondents were married. It is higher compared to the [29] study where 69.3% of participants were single. In the [19] study, 95% of respondents were married, which is similar to the present study. These variations in marital status may reflect different cultural or socioeconomic factors influencing the study populations.

In the present study, 39.6% of the farms had been established for 5–15 years. This aspect was not directly comparable to other studies as similar data were not provided. However, a study in Pakistan reported that 73% of farmers had been in dairy farming for over 10 years [14]. This information can influence farming practices and awareness of aflatoxin management. Training is a crucial factor in improving knowledge and practices. The current study found that 72.6% of participants had not received training. This lack of training is significant, especially when compared to other studies. In central Ethiopia, only a quarter of the farmers had training in dairy farm management [31]. In Northwest Ethiopia, only 27% of dairy farms had training in animal feed handling [33]. These findings indicate a widespread lack of formal training across different regions.

In this study, 89.6% of respondents had heard about aflatoxins, with 59.4% demonstrating good knowledge and 37.7% having satisfactory knowledge. This high awareness contrasts sharply with other studies. For instance, in Northwest Ethiopia, only 6% of dairy farmers could accurately describe feed aflatoxin contamination [33]. Similarly, in Cameroon, only 12% of groundnut farmers were aware of mycotoxins, and none knew about aflatoxins [30]. The discrepancy underscores the relatively better knowledge among participants in the current study, though it also highlights ongoing gaps, particularly regarding the effects of aflatoxins, which 60.4% of respondents were unaware of. In Kenya, knowledge levels varied, with most traders having low (69.8%) or medium (30.2%) knowledge [34]. Educated and female traders were more knowledgeable, highlighting the role of education and gender in knowledge dissemination. This finding aligns with our study's observation that education significantly impacts aflatoxin knowledge scores (*p* = 0.006).

A favorable attitude towards aflatoxin contamination was observed in 94.3% of respondents in the current study. This positive attitude is comparable to findings in Kenya, where socioeconomic and demographic factors did not significantly affect farmers' attitudes towards aflatoxin control [35]. However, in Ethiopia, many producers were not well-informed about moldy cereals' presence in brews [36], indicating a less informed attitude in certain contexts. In Malawi, less than 50% of dairy farmers perceived mycotoxins as risky to humans and dairy animals, reflecting a gap in attitude despite awareness [37]. The generally favorable attitude in the present study suggests a readiness to adopt better practices if accompanied by enhanced knowledge and practical training.

Practice scores in the current study were significantly influenced by education level, with secondary education level respondents reporting the highest practice scores (6.54 ± 1.808). This finding is consistent with results from Cameroon, where good practices were linked to production experience [30]. However, the overall practice levels were concerning, with only 1.9% of respondents exhibiting good practice, 60.4% satisfactory, and 37.7% poor practices. In Kenya, Eldama Ravine subcounty had the highest knowledge scores, suggesting a correlation between knowledge and practice [35]. However, in Malawi, despite awareness, practices to mitigate mycotoxin risks were low, with 60% of dairy farmers unaware that mycotoxins in feed could contaminate milk [37]. This highlights the challenge of translating knowledge and attitudes into effective practices.

## 5. Limitations of the Study

The study did not measure contamination levels using different techniques to quantify aflatoxin amounts in milk and feed, limiting the ability to correlate KAP with actual contamination levels. The sample size was limited and concentrated in a specific geographic area, potentially affecting the generalizability of the findings to a broader population. The absence of longitudinal data prevents the assessment of long-term trends and the effectiveness of interventions over time.

## 6. Conclusion

In conclusion, the study provided higher knowledge of aflatoxins compared to other regions, with significant influence from educational levels. Attitudes towards aflatoxin contamination are generally positive, suggesting potential for improved practices. However, actual practices lag behind, indicating a need for targeted educational programs and practical training. This shows the importance of continuous education and training to bridge the gap between knowledge and practice, ensuring that farmers are equipped not only with the knowledge but also with the practical skills necessary to mitigate aflatoxin contamination effectively. Future interventions should focus on integrating aflatoxin education into broader agricultural training programs and practical workshops, ensuring that farmers at all education levels understand the risks and management strategies for aflatoxin contamination. Additionally, leveraging the generally positive attitudes towards aflatoxin control can help drive the adoption of better practices, ultimately reducing the prevalence and impact of aflatoxins in food and feed supplies as well as minimizing its negative effect on the economy.

## Figures and Tables

**Table 1:** Sociodemographic characteristics of the respondents.

Sociodemographic characteristics	Categories	Frequency	Percent (%)
Gender	Male	85	80.2
	Female	21	19.8

Age	20–30	14	13.2
	30–50	64	60.4
	> 50	28	26.4

Educational level	Illiterate	51	48.1
	Primary	37	34.9
	Secondary	13	12.3
	Tertiary	5	4.7

Dairy farm type	Small-scale (≤ 5)	102	96.2
	Large-scale (> 5)	4	3.8

Occupation	Housewife	13	12.2
	Government employees	1	0.9
	Private employee	20	18.9
	Farmer	68	64.2
	Student	4	3.8

Marital status	Single	9	8.5
	Married	91	85.8
	Divorced	3	2.8
	Widowed	3	2.8

Role in food supply chain	Producer	103	97.2
	Importer	0	0
	Processor	2	1.9
	None of these	1	0.9

Year of farm establishment	5–15	42	39.6
	15–30	40	37.8
	> 30	24	22.6

Training	Yes	29	27.4
	No	77	72.6

**Table 2:** Comparison of mean scores for knowledge, attitude, and practice on aflatoxin among sociodemographic factors.

Variables	Categories	Knowledge score		Attitude score		Practice score	
		Mean ± standard deviation	*p* value	Mean ± standard deviation	*p* value	Mean ± standard deviation	*p* value
Age	20–30	10.43 ± 2.563	0.05	3.71 ± 1.267	0.046	5.64 ± 2.061	0.317
	30–50	12.09 ± 1.604		3.38 ± 0.787		5.78 ± 1.474	
	> 50	11.82 ± 1.362		3.68 ± 0.670		5.61 ± 1.197	

Gender	Male	11.84 ± 1.792	0.346	3.52 ± 0.868	0.886	5.71 ± 1.526	0.694
	Female	11.67 ± 1.713		3.43 ± 0.746		5.76 ± 1.338	

Educational level	Illiterate	11.75 ± 1.481	0.006	3.39 ± 0.695	0.038	5.53 ± 1.419	0.027
	Primary	12.16 ± 1.788		3.54 ± 0.869		5.84 ± 1.214	
	Secondary	10.85 ± 2.444		3.92 ± 0.954		6.54 ± 1.808	
	Tertiary	12.20 ± 1.924		3.20 ± 1.483		4.60 ± 2.302	

Dairy farm type	Small-scale (≤ 5)	11.89 ± 1.659	0.001	3.50 ± 0.830	0.196	5.72 ± 1.360	≤ 0.001
	Large-scale (> 5)	9.50 ± 3.109		3.50 ± 1.291		5.75 ± 3.862	

Occupation	Housewife	11.46 ± 1.761	0.730	3.69 ± 0.751	0.045	6.23 ± 1.013	0.069
	Government employee	10.00 ± 0.00		4.00 ± 0.001		4.00 ± 0.001	
	Private employee	11.25 ± 2.074		3.90 ± 1.071		6.15 ± 1.843	
	Farmer	12.04 ± 1.697		3.37 ± 0.771		5.60 ± 1.405	
	Student	12.00 ± 0.816		3.00 ± 0.001		4.25 ± 0.957	

Marital status	Single	10.22 ± 2.682	0.478	4.00 ± 0.866	0.706	5.33 ± 1.118	0.514
	Married	11.95 ± 1.622		3.46 ± 0.847		5.85 ± 1.505	
	Divorced	12.00 ± 2.000		3.67 ± 0.577		4.00 ± 1.001	
	Widowed	12.00 ± 1.000		3.00 ± 0.001		4.67 ± 0.570	

Role in the food supply chain	Producer	11.82 ± 1.792	0.998	3.49 ± 0.839	0.514	5.71 ± 1.499	0.999
	Importer						
	Processor	11.50 ± 0.707		3.50 ± 0.707		6.00 ± 1.414	
	None of these	11.00 ± 0.00		5.00 ± 0.001		6.00 ± 0.001	

Year of farm establishment	5–15	11.38 ± 2.012	0.277	3.62 ± 0.987	0.338	5.88 ± 1.811	0.371
	15–30	12.10 ± 1.499		3.35 ± 0.770		5.58 ± 1.299	
	> 30	12.04 ± 1.654		3.54 ± 0.658		5.67 ± 1.129	

Training	Yes	11.90 ± 1.819	0.288	3.48 ± 1.056	0.171	5.97 ± 1.742	0.052
	No	11.77 ± 1.761		3.51 ± 0.754		5.62 ± 1.377	

## Data Availability

The questionnaire data used to support the findings of this study are available from the corresponding author upon request.
